# King George III of England and Queen Maria I of Portugal: bipolar disorder and prince regents as common features of their reigns

**DOI:** 10.47626/2237-6089-2021-0315

**Published:** 2023-01-04

**Authors:** M. da Mota Gomes, Lucio Lage Gonçalves, Elie Cheniaux, Antonio E. Nardi

**Affiliations:** 1 Laboratório de História da Psiquiatria, Neurologia e Saúde Mental Instituto de Psiquiatria Universidade Federal do Rio de Janeiro Rio de Janeiro RJ Brazil Laboratório de História da Psiquiatria, Neurologia e Saúde Mental, Instituto de Psiquiatria, Universidade Federal do Rio de Janeiro (UFRJ), Rio de Janeiro, RJ, Brazil.; 2 Instituto de Psiquiatria UFRJ Rio de Janeiro RJ Brazil Instituto de Psiquiatria, UFRJ, Rio de Janeiro, RJ, Brazil.; 3 Universidade do Estado do Rio de Janeiro Rio de Janeiro RJ Brazil Universidade do Estado do Rio de Janeiro (UERJ), Rio de Janeiro, RJ, Brazil.; 4 Academia Nacional de Medicina Brazil Academia Nacional de Medicina (National Academy of Science), Brazil.

**Keywords:** Bipolar disorder, antisocial personality disorder, mental health, political behavior

## Abstract

Humanity is sporadically subjected to leaders with deviant behavior, ego problems, or psychiatric disorders, potentially leading to social instability. Bipolar disorder is not common in all populations, but, coincidentally, studies suggest that it affected two sovereigns that were contemporaries, King George III of England, who died 201 years ago, and Queen Maria I of Portugal, who died 205 years ago. They lived during a time when Europe was in turmoil with the French Revolution and Napoleonic Wars, which also coincided with the rise of psychiatry. Both monarchs were forced to have prince regents rule in their place, due to their emotional decline, and they shared the same medical consultant, Francis Willis.

## Introduction

The mental health of some unusual monarchs, prime ministers, presidents, and political leaders in general has been questioned, and others were unmistakably mentally ill, potentially threatening their nations’ stability.^1^ Indeed, insanity and politics are often intertwined.^2^

As with the general population, much of the insanity in political leaders is influenced by genetics, which is most tragic when these leaders occupy hereditary political positions.

Thus, when monarchs develop a mental illness, several steps are needed to remove them from power. We discuss two such late 18th-century/early 19th-century sovereigns of nations with close political relations.

George III of England, the “Mad King,” died 201 years ago. Four years earlier, Queen Maria I of the United Kingdom of Portugal, Brazil, and the Algarve, the ‘Crazy Queen’, passed away. Both monarchs were 81 years old at the time of their deaths and there were many other coincidences with repercussions for three countries (England, Portugal, and Brazil).

In the late 18th and early 19th centuries, it was not possible to make proper psychiatric diagnoses and treat sidelined sovereigns during their own lifetimes. Both the monarchs discussed here have been assumed to have suffered from bipolar disorder (BPD). They also shared the same attending physician, Francis Willis.^3^

This article raises questions concerning the two monarchs’ mental symptoms and the impact of their physician’s immersion in the medical knowledge of the time. These issues belonged to an age of turmoil in Europe.

## Impacts of the psychological process on the legal and political scene

Monarchs turn their daily exercise into a grand dramatization, in a balancing act of power through their titles, medals, and privileges. As gifts bearing the leader’s image, these monarchic rituals help foster reverence and extend the monarchs’ own personalities, hovering high over their subjects’ heads.^4^

In a monarchy, if psychological instability arises within the court, the natural substitution within the family hierarchy begins to be articulated. Even if the probable succession favors an older child, court dealings may always happen.

Prolonged mental illness in a monarch can have psychological effects on his or her subjects, besides arousing interest among successors and members of the court.

A leader is expected to guide the people, show the way, and determine the nation’s political scenario. Thus, unstable and psychologically compromised behavior with evident loss of control tends to result in the leader becoming discredited and losing the ability to remain in power.

Political leaders are not replaced this way in democratic systems, which require constitutional provisions to ensure substitution in the case of such impediment. Although a leader may appear insane or reckless, more reliable methods to confirm or disprove his or her insanity are required, which was not the case in the late 18th and early 19th centuries, a time when diverse behavioral changes were labeled as madness.

## The “mad” monarchs

### King George III of England

George III (1738-1820, r. 1760-1820) inherited the English throne upon the death of his father in 1751. The American colonies gained independence during his reign, which also witnessed the French Revolution and the Napoleonic Wars, which finally ended with Napoleon’s defeat.^5^

England was shaken by King George’s erratic behavior. Macalpine & Hunter (1969), *apud* Pearce,^3^ state that the king suffered from porphyria, a metabolic disease that can affect the central nervous system. These findings were widely covered by the English press, but the main diagnostic hypothesis today would be bipolar disorder.^3^

During George III’s illness, the Prince of Wales was regularly sent letters by his father’s physicians, reporting on his habits and behaviors. There are several reports of the king’s illness. It was noted in 1788 that “His Majesty had become more peevish than he used to be and is agitated and talking incessantly and incoherently.” Later that month, on December 20, the king’s condition worsened still further, because “H.M became so ungovernable that recourse was had to the strait waistcoat: His legs were tied, & he was secured down across his Breast, & in this melancholy situation he was, when I came to make my morning Enquiries.”^6^

When the king was stricken by “madness” in the summer of 1788, his condition worsened, and he was finally removed from power after a major mental crisis. To control variations in the king’s behavior, physician Francis Willis was summoned to treat his disorder. As his doctors were unable to explain the king’s illness, false stories about his condition spread.^7^

George III was excluded from contact with the people, due to an alleged plot by his son and allies, pressured by a populace with a supposedly insane ruler who was unfit to rule.

The English people appeared to be right, because the first criterion for determining an individual’s insanity is to demonstrate that he is unfit for his job.^8^

The king’s condition worsened over the course of 1788, giving rise to moves to replace him with a prince regent. In November of the same year, he was seriously demented, talking nonstop for hours.^4^ Stories of his mental instability included compliments to a tree as if it were the King of Prussia, causing his physicians to tie him to the bed until he regained calm. Following some improvement, when he was behaving more cogently, he was again deemed fit to rule over his court.^9^

If observed today, this behavioral variation would be considered a sign of bipolarity, an element that would make exercise of political power unacceptable in the eyes of the people.

Political unrest during the reign of George III was not due only to his psychological instability. Legal aspects of the monarchic regime were also present in the government.^7^

In early 1789, the Regency Bill authorizing the Prince of Wales to act as regent was submitted to and approved by the House of Commons. However, George III recovered before the bill was passed by the higher legal and political body, the House of Lords.^9^

The entire search for succession unfolded in a turbulent way in Parliament, with regard to the terms of the regency during the king’s disability. Both of the prevailing political factions agreed that George III’s eldest son and heir apparent would act as regent. One faction was fighting for approval of this legal and political measure by vote in Parliament^10^ (House of Commons and House of Lords).

Meanwhile, the British were worried by the turmoil of the French Revolution, which had overthrown the French monarchy in 1789. George III increased taxes, summoned armies, and suspended the right to habeas corpus and other legal safeguards, thereby exacerbating the political instability.^9^

In 1801, and again in 1804, George III had relapses that further fueled the legal and political turmoil with this psychological component, from 1810 until his death in 1820.^9 , 10^

Contemporary accounts credit Dr. Francis Willis (1718-1807) for facilitating the king’s recovery from his main episode of acute mania in 1788-89, enhancing Willis’ reputation and expanding his clinical practice.

Since Dr. Francis Willis was credited for King George III’s recovery from this serious episode of acute mania in 1788-89, he was summoned to Lisbon to advise on Queen Maria I’s mental health problems. He recommended a moral management policy, psychotherapy, and adequate nutrition rather than medication, and the initial reports were encouraging. However, unlike his treatment of George III, Willis’ role in Maria’s case was merely advisory, and the queen’s prognosis may have been worse than that of George III.^11^

**Figure 1:**
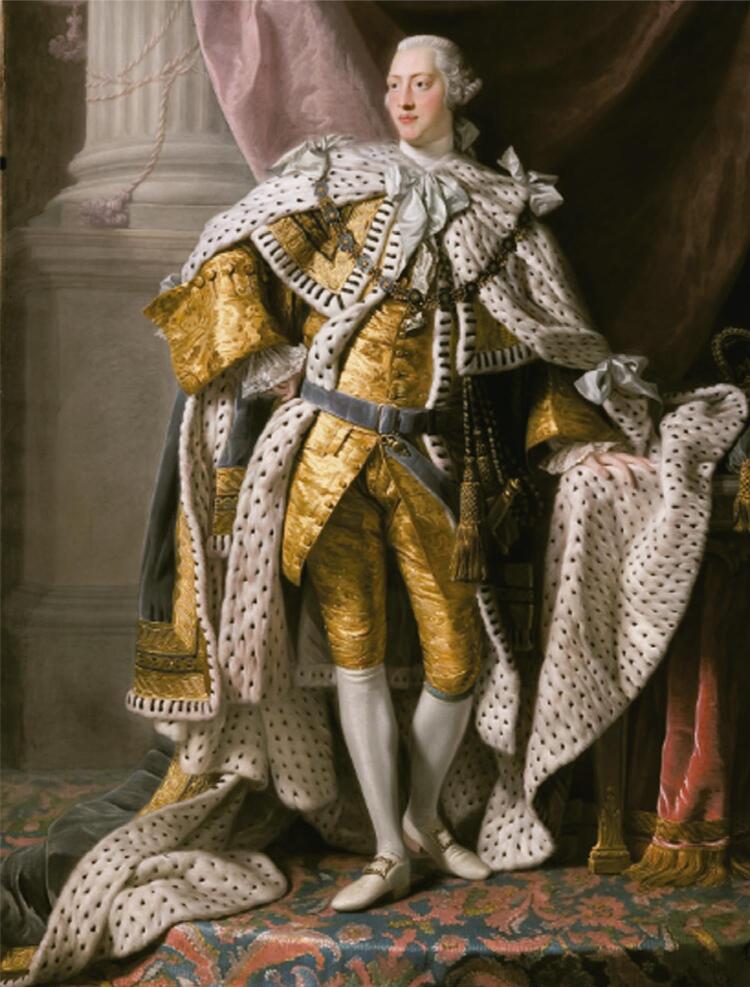
King George III of England. King George III in coronation robes, by Allan Ramsay, circa 1765, in the Art Gallery of South Australia. Public domain.

Willis was awarded his medical degree at the University of Oxford in 1759. He was successful in treating the mentally ill and even ran a private rural asylum in Lincolnshire, where his patients were encouraged to perform manual labor. The clinic’s bucolic rural setting probably contributed to the patients’ recovery. In November 1788, he was summoned to attend King George, whose behavior was becoming increasing erratic. With Willis’ methods, the king’s mental health improved slowly but remarkably ( Figure 1 ).^3 , 12^

## Queen Maria I of Portugal

Maria I (1734-1816, r. 1777-1816) was heir to the throne of Portugal. As a woman, for legal reasons, she could only accede to the throne if she married a Portuguese citizen, so she married her uncle, Infant Pedro of Portugal, in 1760.^5^

In May 1786, the queen’s husband died, and her condition deteriorated further after the death of her eldest son (1788) and her confessor. The alarming news of the French Revolution may have further contributed to the queen’s mental instability. Her mental disorder left the queen unable to deal with state affairs starting in 1792, when her son, who would later become King João VI, officially served as Prince Regent, from 1799 onwards.^5^

The antithesis of Maria I of Portugal was the Marquis of Pombal, a kind of prime minister of Portugal at the time, who tried to have her dismissed when she ascended to the throne. Signs of her dementia appeared two years after the death of her spouse. In 1792, a medical board declared her unfit to rule, transferring the powers of government to her son Dom João (later João VI). In 1799 he received the title of First Regent and later became king in 1816 following his mother’s death.^12^

The impact of a “crazy” ruler, plus the opposition led by Pombal (with whose ideas and actions the queen disagreed) was extremely relevant. She was classified as “crazy” but was probably depressed due to several deaths in the family and that of her friend and confessor Friar Inácio.^13^

The queen was highly popular, and her demise and removal from power must have had a significant impact on her subjects, with whom she had interacted a great deal.

Maria I’s mood swings may explain why, having subjected the Marquis de Pombal to an inquiry to investigate his alleged illicit enrichment and excesses committed in his Ministry, she nevertheless forgave him without further consequences. In August 1781, Pombal was indicted and merited exemplary punishment, but the queen did not order further proceedings due to her serious illness and decrepit state. Queen Maria I issued a decree pardoning the former minister while ordering him to be kept 20 leagues from the court, but maintaining his salary as secretary of state and awarding him the Commendation of St James (São Tiago).^14^

Would such dualities be signs of bipolarity, a mental disorder unknown in the early 19th century? How might these mood swings have impacted Maria I’s decisions as Portuguese sovereign?

From 1792 on, Dom João ruled Portugal as Prince Regent and his mother’s psychological instability no longer interfered in government affairs. In 1807, in response to the French Revolution, which had started in 1789 and was already threatening to affect Portugal, Dom João decided to flee Portugal with his entire court, landing in Brazil in January 1808. Dom João legally transferred the administration of the Kingdom of Portugal to Brazil, which became the kingdom’s official seat.^13^

Maria I and two of her three sisters (Mariana and Dorothea) had similar symptoms of the same mental disorder. Mariana died at the age of 77 in Brazil, and Dorothea at the age of 32. The Queen’s son, Dom João/João VI, also suffered from episodes of melancholy.^11^

**Figure 2:**
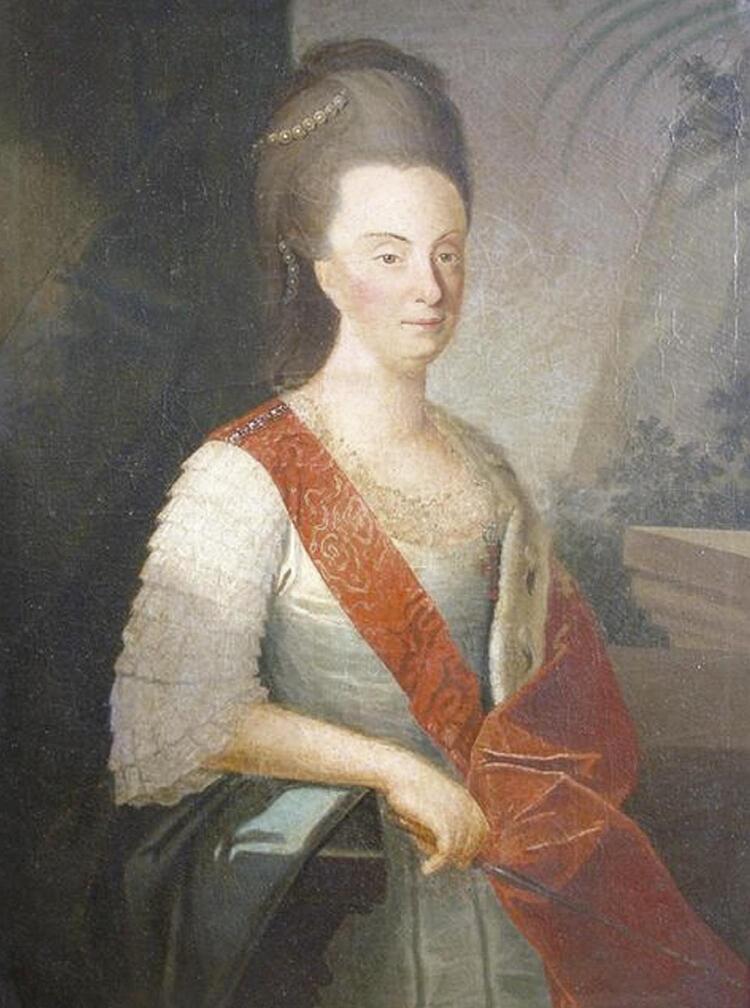
Queen Maria I of Portugal. Portrait of Queen Maria I of Portugal, circa 1780, attributed to Inácio de São Paio. Public domain.

Queen Maria I ( Figure 2 ) became known as Maria the Pious in Portugal and the Mad Queen in Brazil. In 1815, after Napoleon was finally defeated, Brazil ceased to be a Portuguese viceroyalty and became part of the United Kingdom of Portugal, Brazil, and the Algarve. Maria I thus became the first monarch of Brazil. In 1821, the royal family had to return to Portugal, but the king’s eldest son Pedro remained in Brazil. There, the following year, he proclaimed Brazil’s independence from Portugal and became the first emperor of Brazil, Dom Pedro I.

## Conclusions

Bipolar disorder was not known as such in the 18th and early 19th centuries, but both monarchs apparently suffered from this mental disorder, classified as “madness” due to the limitations of prevailing medical knowledge. Regardless of the type of disorder, a monarch’s health or illness is an important psychological factor and thus a predictor of their own stability and that of their kingdom. Psychological, legal, and political factors are intrinsically intertwined in this scenario.

Bipolar disorder affected two sovereigns, contemporaries, who underwent the generic treatment imposed on mental patients at the time by the same attending physician, but with serious limitations to its effectiveness.

Queen Maria I of Portugal had worse prognosis than King George III of England and responded less to specific recommendations from the same physician. Worthy of note is the high degree of consanguinity of the Portuguese royal lineage, which resulted in marked family incidence of mental illness.

In conclusion, with regard to concerns of State, monarchies have an appropriate hierarchy to respond and adjust when the sitting monarch displays mental incompetency, as occurred with George III and Maria I.
